# Home‐based physical activity after treatment for esophageal cancer—A randomized controlled trial

**DOI:** 10.1002/cam4.5131

**Published:** 2022-08-18

**Authors:** Poorna Anandavadivelan, Kalle Malberg, Karin Vikstrom, Sandra Nielsen, Ulrika Holdar, Asif Johar, Pernilla Lagergren

**Affiliations:** ^1^ Surgical Care Science, Department of Molecular Medicine and Surgery Karolinska Institutet Stockholm Sweden; ^2^ Medical Unit Occupational Therapy and Physiotherapy Function Allied Health Professionals, Karolinska University Hospital Stockholm Sweden; ^3^ Department of Surgery and Cancer Imperial College London London UK

**Keywords:** cancer survivorship, esophageal neoplasms, hand grip strength, home‐based exercise, lower extremity strength, physical activity

## Abstract

**Background:**

The treatment of most esophageal cancer patients includes chemo(radio)therapy and extensive surgery, causing physical decline with loss of muscles. This trial aimed to test the hypothesis that a tailored home‐based physical activity (PA) intervention improves muscle strength and mass in patients having undergone curative treatment for esophageal cancer.

**Methods:**

Patients operated for esophageal cancer 1 year earlier were included in a nationwide randomized controlled trial in Sweden in 2016–2020. The intervention group was randomized to a 12‐week home‐based exercise program, while the control group was encouraged to maintain routine daily PA. The primary outcomes were changes in maximal/average hand grip strength measured with hand grip dynamometer and lower extremity strength measured using 30‐second chair stand test and muscle mass measured using a portable bio‐impedance analysis monitor. Intention‐to‐treat analysis was used, and results were presented as mean differences (MDs) with 95% confidence intervals (CIs).

**Results:**

Among 161 randomized patients, 134 completed the study, 64 in the intervention group and 70 in the control group. Compared with the control group (MD 2.73; 95% CI 1.75–3.71), patients in the intervention group (MD 4.48; 95% CI 3.18–5.80) had statistically significantly (*p* = 0.03) improved lower extremity strength. No differences were seen for hand grip strength or muscle mass.

**Conclusion:**

A home‐based PA intervention 1 year after surgery for esophageal cancer improves lower extremity muscle strength.

## Graphical abstract

Patients operated for esophageal cancer 12 months earlier benefited from a home‐based physical activity exercise program. The 12‐week, home‐based exercise program with resistance training showed an improvement in lower extremity muscle strength. Home‐based physical activity in long‐term esophageal cancer survivors may improve rehabilitation.

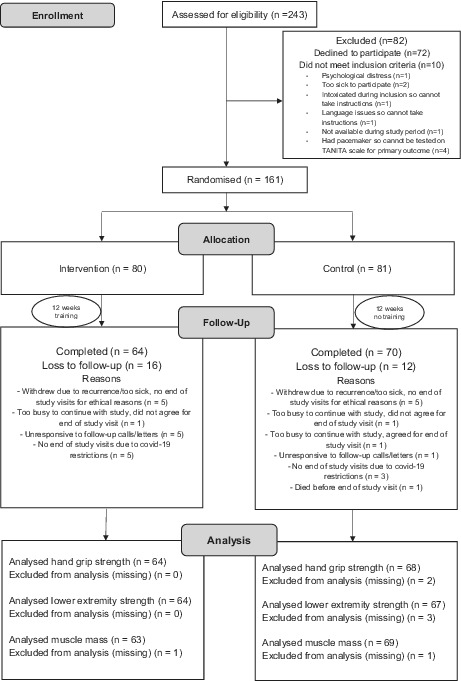

## 1 INTRODUCTION

Esophageal cancer is a debilitating disease with poor overall survival.^1^ In patients selected for curatively intended treatment, that is, mainly surgery (esophagectomy) combined with chemo(radio)therapy, the 5‐year survival is 30%–40%.^2^, ^3^, ^4^ Esophagectomy is an extensive surgery^1^ and the neo‐adjuvant or perioperative chemo(radio)therapy further increases patient burden.^5^, ^6^, ^7^ Most patients face compromised physical function,^8^ not the least due to loss of muscles.^8^, ^9^ There is a great need for well‐designed trials to prove the benefit of novel interventions that may improve physical recovery.^8^ Previous studies in other cancer types show that exercise interventions can improve physical function in patients undergoing active treatment for cancer.^10^ Although physical resistance training after the initial recovery period following treatment for esophageal cancer show promising results of improvement in functional capacity, there is lack of evidence on its role in long‐term survivors.^11^ There is a strong need to test if exercise interventions can improve muscle strength and mass and in turn physical function in long‐term survivors of esophageal cancer. This trial was designed to test the hypothesis that home‐based exercise training in 1‐year survivors of esophageal cancer improves their muscle mass and muscle strength.

## 2 METHODS

### 2.1 Study design and participants

The Physical Activity (PA) study was a Swedish nationwide, two‐armed, multi‐centre randomized controlled trial (RCT) with 1:1 allocation during the study period 2016–2020. Ethical approval was obtained from the Regional Ethical Review Board in Stockholm, Sweden (Dnrs:2015/2142–32; 2016/1696–32/1; 2018/1447–32) and the study was registered with ClinicalTrials.gov, ID: NCT02774551. All procedures conducted in the study were in line with the ethical standards of the 1964 Declaration of Helsinki and its later amendments.^12^ Written informed consent was obtained from all participants. The manuscript was prepared in line with CONSORT 2010 guidelines.^13^

**FIGURE 1:**
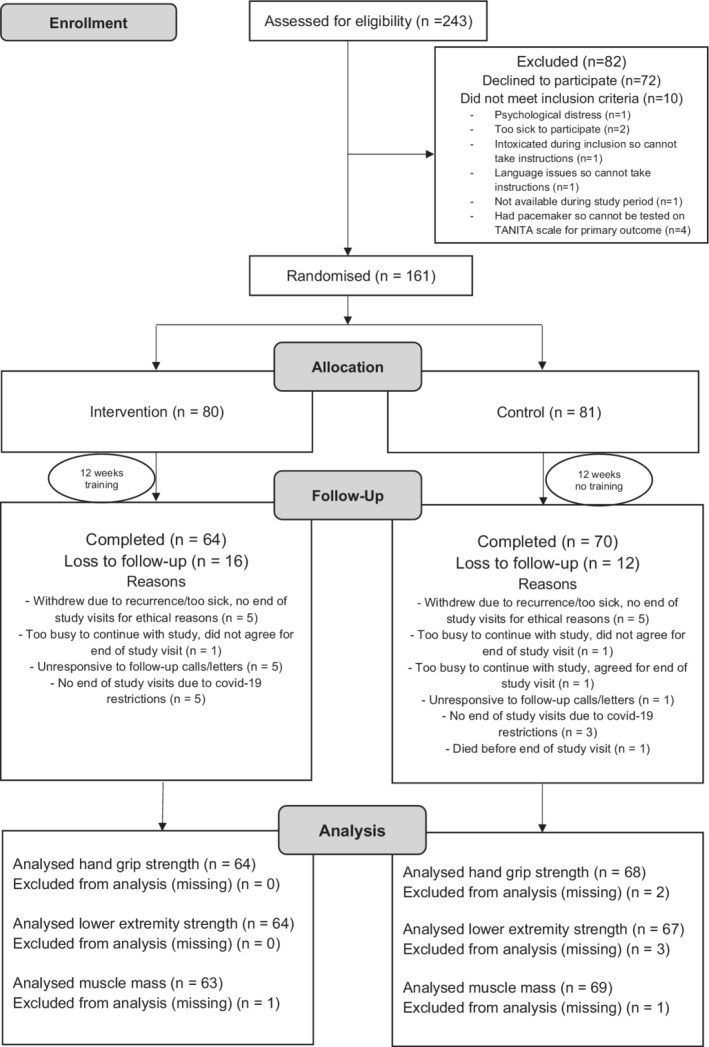
Participant CONSORT flow diagram of the progress through the phases of the Physical Activity study trial for the intervention and control groups regarding the primary outcomes

The study participants were recruited from within the framework of a nationwide and prospective data collection in Sweden, The Oesophageal Surgery in Cancer patients: Adaptation and Recovery study (OSCAR), presented in detailed elsewhere.^14^ The OSCAR study includes all Swedish‐speaking patients with no cognitive dysfunction who underwent curatively intended surgery for esophageal cancer in Sweden from 2013 and onwards and included in the cohort 1 year postoperatively, that is, from 2014 and onwards. The patients were identified through pathology departments at one of the seven university hospitals conducting surgery for esophageal cancer in Sweden. All patients within the OSCAR study were invited to participate in this RCT. Eligibility criteria to be included in the RCT were all patients in Sweden who underwent esophagectomy for esophageal cancer in 2015–2019 included in the OSCAR study 1 year after surgery, that is, between 2016 and 2020. Patients were excluded from the trial if they had physical, psychological or social conditions that prevented participation (Figure 1). Moreover, patients with pacemakers, cochlear or other electrical implants considered unsafe to use the bio‐impedance scale in the trial were excluded for safety reasons.

### 2.2 Randomization

All patients were provided oral and written information about the study at a visit by a research nurse and those who agreed to participate provided consent. The randomization procedure (block randomization) was conducted by an external organization outside the research group (Karolinska Trial Alliance, KTA) to avoid selection bias by anonymising the patient ID. Due to the nature of the intervention, it was not possible to conceal the participants' group allocation to neither patients nor researchers involved. However, the participants' allocation to intervention or control groups was masked in the dataset available for researchers during data analysis. Half of the eligible patients were randomized to intervention with home‐based exercise and half to standard care (control group).

### 2.3 Intervention versus standard care

The intervention consisted of a 12‐week, home‐based exercise training based on previous data.^15^ Firstly, the research nurse suggested 150 min weekly activity of minimum intensity (e.g., walking, swimming, cycling) to the patients. They were as well encouraged a daily lifestyle activity (minimum intensity PA), for example, gardening, vacuuming to contribute to the 150 min, in the absence of aerobic activities. The research nurse introduced 5 simple strength training exercises (Table 1) targeting the major muscle groups with repetitions of 10 and 2 sets to do twice a week. The exercises were modified from the Centers for disease control and prevention, recommendations: strength training for older adults,^16^ the Swedish recommendations for PA in the Prevention and Treatment of Disease for elderly^17^ and for cancer patients.^18^ The exercises were modified considering the average age of the target group and the possibility to conduct them at home. The resistance training was progressive in nature by means of increasing resistance in the band during the 12 weeks if acceptable with the patients and depended on their adaptability to the intervention. The patients tested the exercises guided by the research nurse. Patients were also provided with a pamphlet and/or video link demonstrating the exercises. All patients (intervention group and control group) were informed about the WHO recommendations of physical activities among older people (150 min weekly and strength training 2 times weekly) and their health benefits.

**TABLE 1:** Tailored home‐based strength training for long‐term survivors of esophageal cancer who underwent surgical resection

Exercise	Targeted muscle group
Squats	Leg muscles, muscles in lower body
Wall pushups	Arms, shoulders, chest muscles
Rowing with resistance band	Upper back and shoulder muscles
Shoulder lift with resistance band	Shoulder, upper back muscles, triceps
Hip abduction	Hip abductor muscles

*Note*: All instructions for the exercises were provided in a printed folder and/or video link; Repetitions for each exercise were 10 times per set, rest for 2 min and repeat 10 times again, twice a week; Progressions with resistance band were from easy to medium to hard as tolerated by the patients.

### 2.4 Compliance

The intervention group were followed up weekly by telephone by the research dietician/nurse to encourage adherence to the intervention. They were asked to report if during the past week they had performed the exercises as per the instructions received from the research nurse with the alternatives: (1) Yes, completely (2) Yes, but not all exercises (3) Yes, but only once (4) No, not at all. Compliance was assessed as “yes” (alternative 1 and 2) and as “no” (alternatives 3/4).

### 2.5 Measurements

All measures of muscle strength and muscle mass were assessed objectively by the research dietician/nurse during the home visit both before randomization (baseline) and approximately 12 weeks after randomization.

Muscle strength was assessed using two separate tests, one for hand grip strength and another for lower extremity strength. (1) Hand grip strength was measured using a hydraulic hand dynamometer (Model SH5001 JAMAR, SAEHAN Corporation, Changwon, S. Korea).^19^ Three consecutive measures were taken on the dominant hand. (2) Lower extremity strength was measured using a 30‐second chair stand test, performed according to instructions from the CDC.^20^ Patients were instructed to sit in the middle of the chair with their hands placed on the opposite shoulder crossed, at the wrists and the feet kept flat on the floor. Patients were told to have their back straight and have the arms against their chest. Patients were asked to rise from the chair on hearing the word “go,” to a full standing position, then sit back down again and repeat this for 30 s. Simultaneously, the research nurse/dietitian started the timer on “go” and recorded the number of times the patient came to a full standing position in 30 s. If the patient was over halfway to a standing position when 30 s elapsed, it was counted as a stand. Both these assessments are validated and are well‐established.^21^

Muscle mass was measured using a portable bio‐impedance analysis monitor (TANITA SC240MA), which measures body composition by placing the patient's feet on electrodes that measured resistance to an electrical signal (impedance). The technique is based on the principle that lean tissues have a high water and electrolyte content, and thus provides a good electrical pathway. The resistance measurement relates directly to the volume of the conductor, which is used to determine total body water, muscle mass (fat‐free mass) and fat mass.^22^

Changes in muscle strength were measured as the difference in absolute change in both maximal and average hand grip strength (dynamometer) and functional lower extremity strength (30‐second chair stand test) between baseline and shortly (within 2 weeks) after the 12‐week intervention period. Changes in muscle mass were assessed as the difference in absolute change in muscle mass (portable bio‐impedance analysis monitor) between baseline and shortly (within 2 weeks) after the 12‐week intervention period.

The secondary outcomes, that is, patient reported outcomes, PA levels, and food intake were not included in the present article.

### 2.6 Clinical data

For all included patients, copies of relevant medical records were retrieved from the hospitals, that is, predominantly from the seven university hospitals to provide additional clinical details about the tumor and treatment. These records were assessed according to a detailed predefined study protocol to ensure uniformity in the data collection.

### 2.7 Sample size

The sample size was determined by the power calculations for the muscle strength and mass. Using one‐sided test and alpha at 0.05, sampling ratio 1, superiority margin of 0.001, to test the superiority of the intervention, 160 patients (80 patients per group after adjusting for 5% drop‐outs) were required for 80% power for detecting increase in muscle strength or muscle mass in at least 25% patients in the intervention group and ≤10% patients in the control group. An increase of 3% in muscle mass represented a minimum expected change.^23^

### 2.8 Statistical analysis

Summary statistics for continuous variables (means and standard deviations) and categorical variables (counts and proportions) were presented for both the intervention and control groups. An aggregate of the compliance for all 12 weeks for each individual was obtained by dividing 100/12 for example, 100% compliance if patients have full compliance for all 12 weeks, 92% if they have missed 1 week, 83% if missed 2 weeks, etc, and presented as mean.

All available data were included in the statistical analysis referring to the intention‐to‐treat approach with respect to the original study group assignment and regardless of the patient's participation rate in the exercise intervention. Complete case analysis was used and no attempt to impute missing data was made. Changes in muscle strength and muscle mass were assessed as continuous outcomes. Mean differences (MD) with 95% confidence intervals (CI) were calculated to compare changes between baseline and post‐intervention assessments for the two groups. Owing to limited sample size, randomization might not be sufficient to control for confounding. Therefore, three models were used. In model 1, no confounders were adjusted for, and independent *t*‐tests were performed. In model 2, adjustments were made for age (continuous), sex (male, female), and preoperative body mass index (continuous), which were identified as potential confounders while deciding the study protocol. In model 2, baseline PA level was also identified as a potential confounder originally but was dropped off in the final analysis because it correlated highly with preoperative body mass index. In the final model 3, adjustments were made for variables for which we observed a major difference between the comparison group in Table 2 (postoperative complications, tumor stage, and co‐morbidities) and preoperative body mass index because this variable was statistically significant between the intervention and control groups at baseline in model 2. The level of significance was set at *p* ≤ 0.05. All statistical analysis were performed by an experienced biostatistician (AJ) who followed a pre‐defined protocol and used the statistical software SAS 9.4 (SAS Institute, Cary, NC).

**TABLE 2:** Baseline characteristics of study participants in a Swedish nationwide randomized controlled trial of a home‐based physical activity program in patients treated with surgery for esophageal cancer

Patient and clinical characteristics	Intervention group *n* = 64	Control group *n* = 70
Sex		
Male	56 (87.5)	55 (78.6)
Female	8 (12.5)	15 (21.4)
Average age at operation (years)^a^	67.5 ± 8.3	67.6 ± 7.6
Preoperative body mass index (BMI)^a^	27.3 ± 5.0	26.8 ± 4.6
Baseline physical activity level^b^		
Low	13 (20.6)	17 (24.3)
High	50 (79.4)	53 (75.7)
Charlson co‐morbidity score^b^		
0	33 (52.4)	24 (35.8)
1	18 (28.6)	24 (35.8)
≥2	12 (19.0)	19 (28.4)
Histological sub‐type^b^		
Adenocarcinoma and HGD	55 (87.3)	55 (82.1)
Squamous cell carcinoma	8 (12.7)	12 (17.9)
Tumor location^a^		
Upper and middle esophagus	5 (7.9)	7 (10.4)
Lower esophagus and cardia	58 (92.1)	60 (89.6)
Neoadjuvant therapy^a^		
Yes	51 (81.0)	56 (83.6)
No	12 (19.0)	11 (16.4)
Type of operation^a^		
Minimally invasive	25 (39.7)	25 (37.3)
Hybrid thora/laparoscopic	25 (39.7)	28 (41.8)
Open esophagectomy	13 (20.6)	14 (20.9)
Postoperative tumor stage^a^		
I	30 (47.6)	23 (34.3)
II	18 (28.6)	23 (34.3)
III–IV	15 (23.8)	21 (31.4)
Postoperative complications^a^		
Low grade (CDS 0–II)	45 (71.4)	42 (62.7)
High grade (CDS III–IV)	18 (28.6)	25 (37.3)

*Note*: Results are *n* (%) unless stated.

Abbreviations: CDS, Clavien Dindo Score; HGD, high‐grade dysplasia.

^a^ Results are mean ± SD.

^b^ Missing values <4%.

## 3 RESULTS

### 3.1 Study participants

Patient enrolment was consecutive and lasted from January 2016 to August 2020. A total of 243 survivors from the OSCAR study were assessed for eligibility. Of them, 233 met the inclusion criteria, but 72 declined to participate. The remaining 161 participants (69.1% of those eligible) were randomly assigned to either the intervention group (*n* = 80) or control group (*n* = 81). Of these patients, 28 (17% of those randomized) were lost to follow‐up (16 from the intervention group and 12 from the control group). Hence, 64 patients completed the study from the intervention group and all 64 were included in the final analysis for hand grip strength and lower extremity strength and 63 for muscle mass (missing = 1). Likewise, 70 completed the study from the control group however only 68 for hand grip strength (missing = 2), 67 for lower extremity strength (missing = 3) and 69 for muscle mass (missing = 1) were included in the final analysis. The progress of all participants during the trial is shown in the flow diagram (Figure 1) adapted from the CONSORT 2010 statement.^13^

Patient characteristics are presented in Table 2. Most participants in both the intervention and control groups were males and had almost the same average age at the date of surgery. The PA levels at baseline were similar between both groups. The main histological type in both groups was adenocarcinoma. Most of the patients received neo‐adjuvant therapy in both groups.

**FIGURE 2:**
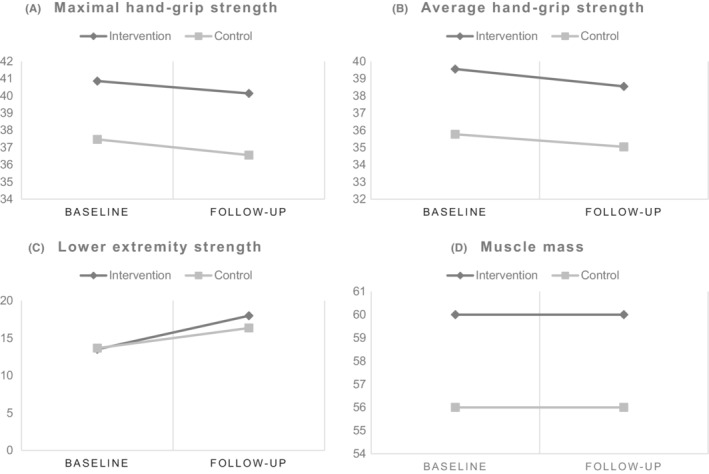
Panel graph showing changes in means of the primary outcomes. (A) Maximal hand grip strength; (B) Average hand grip strength; (C) Lower extremity strength; (D) Muscle mass assessed between 1 year after surgery for esophageal cancer (baseline) and after approximately 12 weeks of physical activity‐based intervention (follow‐up)

The compliance rate in the intervention group was 91%. The changes in the means between baseline and post‐intervention, comparing the intervention with control group for all the primary outcome measures are presented as a panel graph in Figure 2.

### 3.2 Hand grip strength

Among the 70 patients with completed measures in the control group, two patients had missing values for hand grip strength and hence were not included in the final analysis (Figure 1). Both the maximal and average hand grip strength showed a decline post‐intervention for both the groups (Figure 2). The point estimates suggested a more marked decline in maximal hand grip strength for the control group (MD: −0.85; 95% CI: −2.03 to 0.32) than the intervention group (MD: −0.72; 95% CI: −1.86 to 0.43) as per model 1, and models 2 and 3 showed similar results (Table 3). In contrast, the point estimates indicated a worse decline in average hand grip strength for the intervention group (MD: −1.01; 95% CI: −2.28 to 0.26) than the control group (MD: −0.62; 95% CI: −1.79 to 0.56) as per model 1, and models 2 and 3 showed same results (Table 3). However, none of these MDs were statistically significant (Table 3).

**TABLE 3:** Differences in primary outcomes between baseline and post‐intervention for the two allocation groups of the Swedish nationwide randomized controlled trial of a home‐based physical activity program in patients treated with surgery for esophageal cancer presented as per the three statistical models

Primary outcomes	Intervention group		Control group		
Model 1	*n*	MD (95% CI)	*n*	MD (95% CI)	*p* value
Maximal hand grip strength	64	−0.72 (−1.86 to 0.43)	68	−0.85 (−2.03 to 0.32)	0.87
Average hand grip strength	64	−1.01 (−2.28 to 0.26)	68	−0.62 (−1.79 to 0.56)	0.65
Lower extremity strength	64	4.48 (3.18 to 5.80)	67	2.73 (1.75 to 3.71)	0.03*
Muscle mass (kg)	63	0.14 (−1.34 to 1.61)	69	0.33 (−0.83 to 1.48)	0.84
Model 2					
Maximal hand grip strength	64	−0.67 (−2.14 to 0.80)	68	−1.04 (−2.40 to 0.32)	0.67
Average hand grip strength	64	−1.13 (−2.68 to 0.42)	68	−0.89 (−2.32 to 0.55)	0.79
Lower extremity strength	64	4.70 (3.31 to 6.09)	67	2.49 (1.20 to 3.79)	0.01**
Muscle mass (kg)	63	0.35 (−1.17 to 1.87)	69	0.33 (−1.07 to 1.74)	0.99
Model 3					
Maximal hand grip strength	64	−0.50 (−1.85 to 0.85)	68	−0.95 (−2.15 to 0.26)	0.61
Average hand grip strength	64	−0.78 (−2.18 to 0.63)	68	−0.58 (−1.83 to 0.67)	0.83
Lower extremity strength	64	4.42 (3.14 to 5.71)	67	2.32 (1.16 to 3.47)	0.01*
Muscle mass (kg)	63	0.36 (−1.02 to 1.75)	69	0.36 (−0.87 to 1.58)	1.00

*Note*: *n* is number of patients with completed measures included in the final intention to treat analysis in both groups; MD is differences in means between baseline and post‐intervention measures for both groups; Model 1 is unadjusted; Model 2 is adjusted for pre‐defined confounders age, sex, and preoperative body mass index; Model 3 is adjusted for postoperative complications, tumor stage, co‐morbidities, and preoperative body mass index; hand grip strength was measured using a hydraulic hand dynamometer; lower extremity strength by means of a 30‐s chair stand test; muscle mass was measured using a portable bio‐impedance analysis monitor.

Abbreviations: CI, Confidence intervals; MD, Mean differences.

* Significant at *p* ≤ 0.05.

** Significant at *p* ≤ 0.01.

### 3.3 Lower extremity strength

From those who had completed measures in the control group (*n* = 70), three patients had missing values for the 30‐second chair stand test and were excluded from this analysis (Figure 1). The changes in means between baseline and follow‐up showed an increase in lower extremity strength measured using the 30‐second chair stand test for both the groups (Figure 2). The MDs between baseline and follow‐up were 4.48 (95% CI: 3.18 to 5.80) for the intervention group and 2.73 (95% CI: 1.75 to 3.71) for the control group as per model 1 and were statistically significantly higher for the intervention group (Table 3). These differences were statistically significantly higher for the intervention group compared with the control group also according to model 2 and model 3 (Table 3).

### 3.4 Muscle mass

Among the participants with complete end of study measures in the intervention group (*n* = 64) and control group (*n* = 70), missing values were observed for (*n* = 1) and (*n* = 2) respectively and were thus not included in the analysis for muscle mass (Figure 1). Changes between baseline and post‐intervention means of muscle mass showed a slight increase for both groups (Figure 2). The control group had more increase in muscle mass than the intervention group in model 1 (MD: 0.14; 95% CI: −1.34 to 1.61 for the intervention group and MD: 0.33; 95% CI: −0.83 to 1.48 for the control group), but not in models 2 or 3 (Table 3).

## 4 DISCUSSION

This trial demonstrated that a specially designed 12‐week home‐based PA program improved lower extremity muscle strength among patients having undergone surgery for esophageal cancer 1‐year earlier, while no influence on hand grip strength or muscle mass was identified.

Some methodological strengths and weaknesses of the trial should be considered. A major strength was the nationwide multicentered design that ensured that all eligible patients were identified yielding a high enrolment rate. Adherence to a rigorous protocol enabled homogeneity in the recruitment process also uniformity and completeness of the data collected. Moreover, a comprehensive variable list with patient and clinical data allowed for adjustment for predefined confounders and variables observed to vary between intervention and control groups at baseline. Another notable strength is the unique home‐visit design for data collection by the research nurse at baseline and research dietician at follow‐up, which aided increased completeness and reduced missing values at both time points. One potential limitation may have been the exclusion of patients not willing to start a PA intervention may present a potential for selection bias. In addition, a slightly higher attrition rate in the intervention group than the control group also may have contributed to some extent of selection bias. Another limitation is that although preoperative BMI was adjusted for in the analysis, unknown confounders such as pre‐operative muscle mass and distribution of body fat among patients may have still differed between the groups but unaccounted for in the analysis but the RCT design may help reduce these imbalances. In addition, the higher drop‐out rate (17% of those randomized) than assumed in the original sample size calculated is another limitation in the present study.

To our knowledge, this is the first RCT to examine the effectiveness of a home‐based PA intervention in long‐term survivors of esophageal cancer. The results cannot be directly compared to other exercise trials among post‐operative esophageal cancer survivors since they were not conducted at the same time point after surgery. In other studies testing exercise interventions after esophageal cancer surgery, the intervention was either multidimensional including both PA and nutritional components or conducted immediately after surgery.^24^, ^25^, ^26^ The present study included a unidimensional intervention of only PA and recruited patients at 1‐year following surgery. This time point was selected because the patients are usually sufficiently recovered to have the motivation and strength to participate in the trial. The main finding was an improvement in lower extremity muscle strength or strength of leg muscles (quadriceps muscle group) in the intervention group. Since the chair stand test requires both strength and endurance, this test is a qualified and convenient measure of strength in general.^21^ On the other hand, the study did not reveal any consistent improvement in favor of the intervention group in hand grip strength more than a non‐significant trend favoring the intervention group in relation to maximal grip strength. This may be explained by an only moderate correlation of grip strength with strength in other body compartments.^21^ The resistance training performed in this study aimed at strengthening the major body muscles, including the leg muscles, and had less focus on arm muscles. The results from our study also did not favor an increase in muscle mass in the intervention as measured by BIA, which was chosen considering the convenience of its portability and its suitability to the design of the trial.^21^ However, these results might have been biased because BIA does not measure muscle mass directly, but instead derives an estimate of muscle mass based on whole‐body electrical conductivity.^27^, ^28^, ^29^, ^30^ In addition, BIA measurements can be influenced by hydration status of the patient.^29^, ^31^ A validation of the portable bio‐impedance analysis monitor (TANITA SC240MA) used in this RCT compared with computer‐tomography scans from patients with upper gastrointestinal cancers is underway and expected to throw more light on the potential for measurement bias. It is also unclear if the intensity of the exercises and their progression as well as duration of the program were optimal to identify a difference in arm muscle strength and muscle mass. Another consideration is that the control group received no comparison treatment and were not blinded to the intervention. This might have led patients to test the intervention or an alternate intervention with the thought that the intervention is beneficial.

A distinctive strength of our trial was the home‐based exercise intervention, that was designed to personalize the intervention according to patient specific considerations such as their average age, invasive treatment history and a nationwide recruitment. The intervention was designed to be simple, safe, easy to perform, monitored from distance, and inexpensive. Home‐based exercise programs show promising potential for effective improvement of symptoms, exercise capacity, and quality of life in patients with lung,^32^, ^33^, ^34^ breast,^35^, ^36^ colorectal,^37^, ^38^, ^39^ and prostate cancer.^40^, ^41^ Patients express strong preference for home‐based exercises in oncology settings owing to removed costs and travel to an exercise facility, and the flexibility to accommodate competing commitments.^42^, ^43^, ^44^ Patients' preference towards participating in home‐based exercise programs are mainly owing to the ability to self‐manage, feeling comfortable exercising without qualified supervision, and a desire for autonomy.^43^, ^44^ Although prehabilitation and rehabilitation have gained significant attention since their incorporation in the enhanced recovery programs in patients being treated for esophageal cancer, studies testing the effectiveness of home‐based interventions are lacking. Ours is the first trial to test a tailored home‐based intervention program in esophageal cancer survivors, a patient group with high requirements for rehabilitation after having undergone one of the most invasive surgical treatments known for cancer.

The long and incomplete recovery with substantial limitations in long‐term recovery in postoperative survivors of esophageal cancer is a concern for patients, healthcare, and society. Most patients need many follow‐up appointments with healthcare during several years, and the limited availability of interventions that can help these patients is a great concern. Thus, the focus on the role of a physical training program after surgery for esophageal cancer in improving muscle strength, which is a major determinant of physical recovery, is imperative. The results of this trial, testing the intervention of a tailored 12‐week home‐based PA program contribute to the development in the post‐operative management of these patients. The results show promising potential for encouraging patients to be physically active after surgery and in turn enhance the rehabilitation and provide a novel and robust tool for better recovery. An improved recovery would decrease the burden on healthcare by increasing patient self‐care and reducing the need for further or extra appointments. A research partnership group consisting of patients operated on for esophageal cancer were presented with the results of the present study and inputs from their interpretation of the results are included in the discussion above.

In conclusion, this nationwide multicenter RCT demonstrated that a tailored home‐based PA intervention induced improvement in lower extremity muscle strength among esophageal cancer patients who underwent surgery for esophageal cancer 1‐year earlier. Although further investigations are warranted to determine the optimum intensity of the intervention and potential measurement bias, the results should be enough for encouraging patients to be physically active after surgery in the longer term to improve the recovery.

## FUNDING INFORMATION

This work is supported by the Swedish Cancer Society 180685, the Swedish Research Council 2017–01744, the Swedish Research Council for Health, Workinglife and Welfare 2017–00812, The Cancer Research Funds of Radiumhemmet 171103 and Region Stockholm (ALF) LS 2018–1157. Pernilla Lagergren is supported by the NIHR Imperial Biomedical Research Centre (BRC) for her position at Imperial College London, London, United Kingdom.

## CONFLICT OF INTEREST

The authors declare no conflict of interest.

## ETHICAL APPROVAL STATEMENT

Ethical approval was granted by the Regional Ethical Review Board in Stockholm, Sweden (Dnrs: 2015/2142–32; 2016/1696–32/1; 2018/1447–32).

## CONSENT TO PARTICIPATE

Written and oral information was given, and informed consent was collected from all participants by the research nurse before the randomization envelope was opened.

## TRIAL REGISTRATION

ClinicalTrials.gov, ID: NCT02774551. Registered 28 April 2016—Retrospectively registered, https://clinicaltrials.gov/ct2/show/NCT02774551. Reasons for retrospective registration—Unaware of prospective registration.

## Data Availability

The data sets generated and/or analyzed in the current study will not be publicly available due to the ethical review act but will be available from the principal investigators of the study on reasonable request.
